# Learning Medical Materials From Radiography Images

**DOI:** 10.3389/frai.2021.638299

**Published:** 2021-06-18

**Authors:** Carson Molder, Benjamin Lowe, Justin Zhan

**Affiliations:** Data Science and Artificial Intelligence Lab, Department of Computer Science and Computer Engineering, College of Engineering, University of Arkansas, Fayetteville, AR, United States

**Keywords:** computer vision, material analysis, convolutional neural networks, siamese neural networks, image classification, medical imaging, radiography

## Abstract

Deep learning models have been shown to be effective for material analysis, a subfield of computer vision, on natural images. In medicine, deep learning systems have been shown to more accurately analyze radiography images than algorithmic approaches and even experts. However, one major roadblock to applying deep learning-based material analysis on radiography images is a lack of material annotations accompanying image sets. To solve this, we first introduce an automated procedure to augment annotated radiography images into a set of material samples. Next, using a novel Siamese neural network that compares material sample pairs, called D-CNN, we demonstrate how to learn a perceptual distance metric between material categories. This system replicates the actions of human annotators by discovering attributes that encode traits that distinguish materials in radiography images. Finally, we update and apply MAC-CNN, a material recognition neural network, to demonstrate this system on a dataset of knee X-rays and brain MRIs with tumors. Experiments show that this system has strong predictive power on these radiography images, achieving 92.8% accuracy at predicting the material present in a local region of an image. Our system also draws interesting parallels between human perception of natural materials and materials in radiography images.

## 1 Introduction

Computer vision, the study of using computers to extract information from images and videos, has become embedded in new, broad medical applications due to the high accuracy that deep learning models can achieve. Recent deep learning models have shown to be effective at solving a variety of vision tasks in medical image analysis like analyzing chest X-rays (Irvin et al., 2019; Wang et al., 2019), segmenting brain scans (Lai et al., 2019), and annotating pressure wounds (Zahia et al., 2018).

However, such deep learning models are greatly affected by the quality of the data used to train them and often sacrifice interpretability for increased accuracy. A lack of quality data, especially in expert domains like medicine, limits the possible tasks that computer vision can be used for. One such task, material analysis, examines low-level, textural details to learn about the textural and physical makeup of objects in images. To make this task feasible without relying on experts to create hand-crafted textural datasets, existing datasets need to be augmented to encode textural knowledge.

**FIGURE 1:**
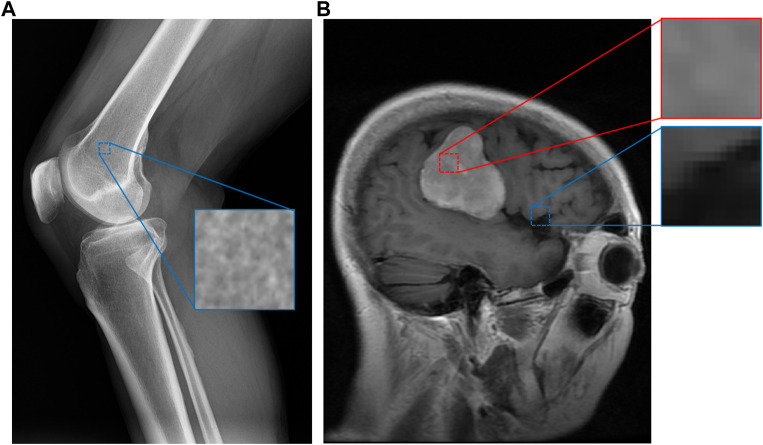
An example of image patches taken from a knee X-ray **(A)** and a brain MRI with a tumor **(B)** (Cheng, 2017). Although some categories, such as bone, have apparent material distinctions, others such as tumor (in red) and healthy (in blue) brain tissue may not have obvious material differences. However, our system can still discern these differences since the images are expertly labeled, while assigning smaller perceptual distances (similarity) between pairs of categories more similar to each other.

Medical images contain a great amount of textural data that has been underexplored. Intuitively, different regions of a medical image exhibit low-level characteristics that imply what kind of material is present in a portion of an image. Figure 1 demonstrates this for a knee X-ray and brain MRI. In this example, a “spongy” section of an X-ray image appears to indicate that the section contains bone, while a brighter region of a brain MRI indicates the presence of a tumor. Many medical image datasets capture such regions of textural interest but do not explicitly encode these textures. For example, brain MRI datasets often include segmentation masks for brain tumors (Cheng, 2017; Schmainda and Prah, 2018) that encode these regions, but without explicit textural context.

While these masks delineate the region where a tumor resides in an image, they give no textural information about the tumors themselves. To obtain this textural information, one must either hire experts to create a dataset of such textures, or leverage these pre-existing annotations in a way that automatically draws out their relationships with the underlying textures and materials. We propose a method to achieve the latter.

In this paper, we introduce a method to analyze medical radiography images with or without such generic annotations to generate a dataset of image patches representing different textures found in medical images. Our method additionally learns an encoding of the relationship between the textural categories in these images and generates a set of machine-discovered material attributes. These material categories and attributes are then used to classify textures found within medical images both locally and over an entire image. Finally, we evaluate our method on a composite dataset of knee X-rays and brain MRIs, observing the attributes learned while also examining how the network automatically performs knowledge transfer for textures between different image modalities.

Our method has the following novel contributions. First, we propose a method to automatically generate a medical material texture dataset from pre-annotated radiography images. Second, we propose a neural network, D-CNN, that can *automatically* learn a distance metric between different medical materials without human supervision. Third, we upgrade MAC-CNN, a material analysis neural network from prior work (Schwartz and Nishino, 2020), to use the ResNet (He et al., 2015) architecture, which maintains its high accuracy while having greater scalability to deeper layers.

The remainder of the paper is structured as follows. In Section 2, we discuss the methodology of our system. In Section 3, we evaluate how our system performs on the composite dataset of knee X-rays and brain MRIs. Finally, in Section 4, we evaluate related works and conclude.

## 2 Materials and Methods

At a high level, our approach uses two convolutional neural network (CNN) architectures to predict the materials that appear in small image patches. These image patches are sourced from full radiography images. For material categories that require expertise to properly label, such as brain tumor tissue in a brain MRI, the patch’s material label is sourced from an expert mask. For more recognizable materials, such as bone and the image background, these labels are sourced automatically based on a region’s average brightness.

The CNNs learn these material classifications while respecting an embedding that encodes the relative difference of pairs of categories, analogous to word embeddings in natural language processing. The system’s material category classification for each image patch is a *K*-long vector where *K* is the number of material categories to be classified, and the system’s material attribute classification is an *M*-long vector where *M* is the selected number of material attributes to be discovered.

To ensure our network is using accurately categorized data, we introduce a thorough patch generation and categorization process on expertly annotated images in Section 2.1. Then, the process to learn the perceptual distances between material categories and encode them in a distance matrix is discussed in Section 2.2. In Section 2.3, we present the discovery process for another matrix that encodes both the material categories’ distances stored in the distance matrix and a new set of material attributes. Finally, in Section 2.4, we introduce the MAC-CNN, which uses this matrix to categorize local image patches into material categories and material attributes. A summary of the notations used is presented in Table 1.

**TABLE 1:** Summary of notations.

Notation	Definition
*T*	Mask tolerance for a given patch
$\bar{B}$	The average brightness value of a given patch
${\bar{B}}_{min},{\bar{B}}_{max}$	The minimum and maximum average brightness allowed
${\bar{B}}_{0}$	The maximum average brightness for the null class
$C_{i}$	The set of patches of category *i*
*N*	The number of patches generated
*k*, *K*	The number of material categories (human)
*m*, *M*	The number of material attributes (generated)
γ	Weight hyperparameter for minimization objectives
Θ	Network parameters
$\Theta^{*}$	Optimized network parameters
$X_{r}$	Set of reference images
$X_{c}$	Set of comparison images
$f\left(x_{n},x_{c}\right)$, $\hat{y}$	D-CNN prediction on reference and comparison sets
*Y*	True similarity value for reference and comparison patch
p	D-CNN vector of binary similarity decisions
D	K×K distance matrix between material categories
A	K×M material category/attribute matrix
q(p;A)	Gaussian kernel density estimate of A at point *p*
β(p;a,b)	Beta distribution with parameters *a*, *b* at point *p*
$A^{*}$	Optimized A matrix
X	Training set of image patches for MAC-CNN
*T*	Pairs $\left(x_{i},y_{i}\right)$ of the set X
$x_{i}$	Raw feature vectors of image patch *i*
$y_{i}$	One-hot encoded label of image patch *i*
$f\left(x_{i}\right)$	MAC-CNN prediction on image patch $x_{i}$
f(T)	Equivalent to $f\left(x_{i}\right)$ but while also considering label $y_{i}$

### 2.1 Patch Selection and Categorization

The first component of the system is selecting and categorizing patches from the medical images so that every patch corresponds highly to its assigned category. Since images vary widely within medicine, such as the differences between X-rays and MRIs, it is important to normalize the images in such a way that the content and annotations are preserved while removing variations that may mislead the system.

Each specific image mode or dataset may use a different approach to patch generation depending on the nature of the source data. The following steps are used to generate patches of background, brain, bone, and tumor categories, but this system can be used to generate image patches in many different medical applications.

To generate the medical-category image patches used to evaluate the system, the first step is to invert negatives (images where the brightest regions indicate dark areas). Then, each image’s raw features are normalized to the range [0,1], and Algorithm 1 is used to generate patches.

**Algorithm 1:** Patch categorization procedure

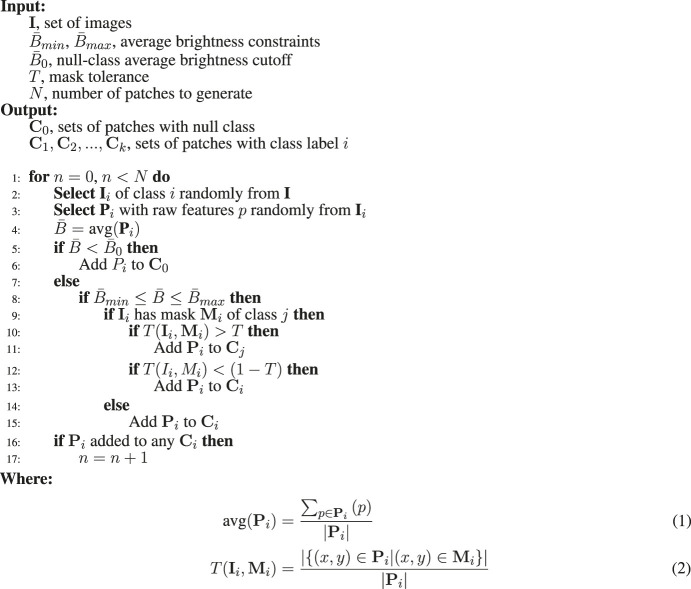

Some images may have expertly annotated masks—like a brain tumor in an MRI. Other images—like the knee X-rays in our experiment—may lack masks and labeling, but the categories sought to be analyzed are simple enough to be assumed. This reduces the detail of the dataset, but still yields useful categories for training which may even be applicable in other image modes. We call material categories that are expertly annotated (such as “tumor”) *expert categories*, while non-annotated material categories (like “bone” for the knee X-rays) are called *naïve categories* since the naïve assumption is made that the average brightness of an image region corresponds to its category.

A third type of material category, the *null category*, corresponds to a category that does not contain useful information, but when isolated can improve the model’s ability to learn the other categories. For the cases of X-rays and MRIs, the null category is derived from the image background.

We believe that brightness constraints are a useful way to extract naïve categories in most cases. Generally, extremely bright regions and dark regions lack interesting texture data—for example, the image background. Meanwhile, moderately bright regions may contain some textural information of interest.

For instance, in identifying brain tumors, gray matter tissue, which may not be annotated with a mask, is not as significant as tumor tissue. However, separating gray matter textures from the background, which is much darker, allows for a classifier to make more specific predictions by preventing it from learning that background regions correspond with gray matter. Additionally, when using multiple image modalities with distinct categories to build a dataset, separating the dark background prevents an overlap in each category’s texture space.

Although we use brightness constraints, other constraints could be used depending on the imaging modality. For example, with a set of RGB color images, a set of constraints could be created from the average value of an RGB color channel.

To generate a material patch from a selected region of an image, the first step is to calculate the average brightness of the region using Eq. 9, which is the sum of all the region’s normalized raw feature values divided by the number of raw features. The constraints ${\bar{B}}_{min}$, ${\bar{B}}_{max}$, ${\bar{B}}_{0}$, and *T* in Algorithm 1 can be altered at run time to create better-fitting categories.

For expert categories, like “tumor”, that are defined by a mask within the image, the patch generation process needs to ensure that a large enough percentage of the region is within the mask. This value is defined as the mask tolerance *T*, presented in Eq. 10. This value is included to avoid categorizing regions that are on the mask boundary, which may confuse the training of the system. We define a small value of T>0 since it allows for patches that intersect categories while still avoiding ambiguity. This increases the pool of eligible image patches, introduces variance to reduce overfitting, and allows for smaller masks (like for pituitary tumors, which are generally small) to be represented in the patch set.

For any expert category patch, at least (1−T)  ×  100 percent of the patch’s source region is inside the mask boundary. For any naïve category patch, at most T  ×  100 percent of the mask is allowed to be within the patch’s source region.

To further normalize the patches, we also introduce the average brightness constraints ${\bar{B}}_{min}$, ${\bar{B}}_{max}$, and ${\bar{B}}_{0}$. Since each patch raw feature is normalized to the range [0,1], the average brightness constraints are likewise constrained to [0,1]. First, if a region has an average brightness $\bar{B}<{\bar{B}}_{0}$, the region’s patch is automatically added to the null category. For another patch to be included in the dataset, its average brightness must fall within the range $\left[{\bar{B}}_{min},{\bar{B}}_{max}\right]$.

Using the above constraints, for each iteration of Algorithm 1, a random image in the set is selected, and within that image, a random point (x,y) from a set of points spaced *p* pixels apart is selected. For the selected point, patch $P_{i}$ is spliced from a 32×32 section of the image below and to the right of (x,y). This patch is evaluated against the constraints to determine if it is eligible to be included in the patch set and what category it belongs to. If the image has a mask, the patch is categorized into the mask or non-mask category based on the mask tolerance value. Patch $P_{i}$ is added to its assigned category set $C_{i}$ if it meets the constraints.

The generation process ensures every saved patch originates from a unique point, meaning there are no duplicate patches in the dataset. Additionally, different image types containing different categories may use different constraint values when generating patches. The final patch set is used to form training, validation, and test datasets for both of the CNNs in the following sections.

### 2.2 Generating a Similarity Matrix for Material Categories

This section introduces a novel Siamese neural network, the *distance matrix convolutional neural network* (D-CNN), that learns to make similarity decisions between image patches to produce a distance matrix D that encodes the similarities between pairs of material categories.

The D-CNN works by making binary similarity decisions between a reference image patch of a given category and a comparison patch of a different or the same category. This network assists in evaluating expert categories since it is effective compared to human similarity decisions on naïve categories, while not requiring the manual annotation necessary for humans.

**FIGURE 2:**
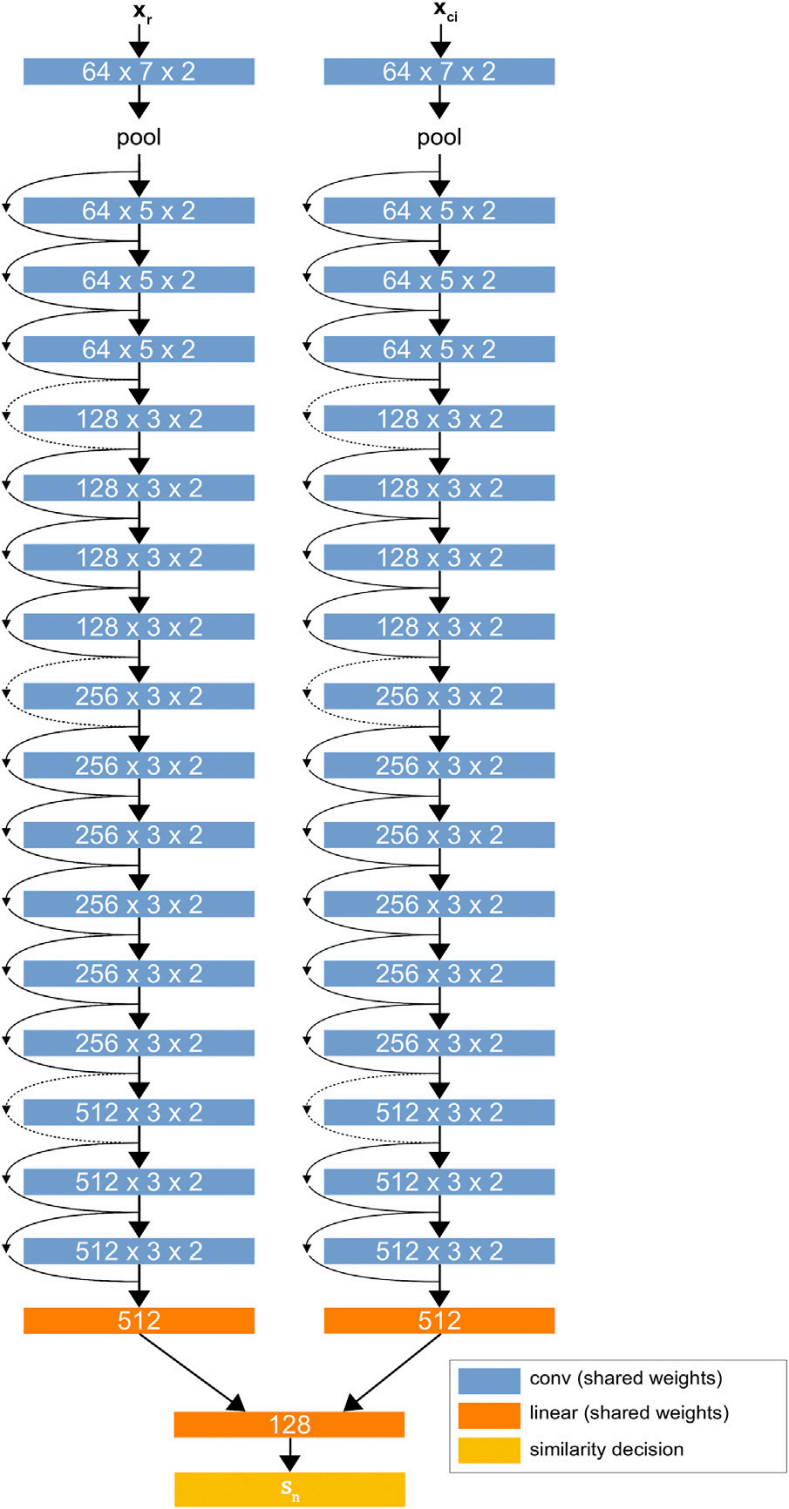
The D-CNN architecture. The two ResNet34 (He et al., 2015) networks share the same weights, forming a Siamese neural network. The linear layers at the end of the network find a difference between the two networks’ values for each patch and give a binary similarity decision $s_{n}$ based on this difference. The goal of training the D-CNN is to maximize its ability to make correct similarity decisions.

The network architecture is based on a modified version of ResNet34 (He et al., 2015) with custom linear layers that perform pairwise evaluation between patches.^1^ Figure 2 shows the D-CNN network architecture. The network is trained on a large dataset of greyscale image patches, each having raw feature vectors $x_{i}$.

The purpose of the D-CNN is to obtain binary similarity decisions $s_{n}={\left\{0,1\right\}}^{n}$ between a reference image and each of a set of *n* images representing each class in the dataset. The Siamese D-CNN does this without human supervision, using a dataset with *k* material categories C={1,2,…,k}. The dataset is divided into batches of reference images $X_{r}$ that are each associated with comparison images $X_{c}$ of every class $c_{i}\in C$. For each sample, the D-CNN is provided a set of k+1 image patches, with the reference image patch $x_{r}\in X_{r}$ having class $c_{i}$ and the *k* comparison image patches $\left\{x_{c_{1}},x_{c_{2}},\ldots ,x_{c_{k}}\right\}$ having unique classes in shuffled order.

A single pass through the D-CNN consists of the reference image $x_{r}$ being paired with one of the comparison images $x_{c_{i}}$. Each patch is sent through the D-CNN’s convolutional layers with the same weights, and the two convolutional outputs are compared in the linear layers. The D-CNN returns $\hat{y}=0$ if it evaluates that the paired images are of the same class or $\hat{y}=1$ if it evaluates that the paired images are of different classes. This process repeats with $x_{r}$ and each of the comparison images $x_{c_{i}}$.

For a D-CNN with network parameters Θ, and predictions $\hat{y}=f\left(x_{r},x_{c};\Theta \right)$ with corresponding similarity decision labels *y*, the training process can be formalized as the minimization problem described in Eq. 1.

$$\Theta^{\text{*}}=\underset{\Theta }{\text{argmin}}\underset{\hat{y},y}{\sum }-\left(y\text{ln}\left(\hat{y}\right)+\left(1-y\right)\text{ln}\left(1-\hat{y}\right)\right)$$ (1)

The minimization term represents the cross-entropy loss between the D-CNN’s predicted value on the comparison between image sets $X_{r}$ and $X_{c}$, and the actual values of the similarity decisions between the two sets. Minimizing this term helps the D-CNN more closely fit the target function, which makes it more accurately evaluate if two image patches are of the same or different material categories.

We note that we selected cross-entropy loss despite many Siamese neural network models using triplet loss (Chechik et al., 2010) in their minimization objective. Triplet loss is useful for tasks like facial recognition (Schroff et al., 2015), where classes cannot be represented in a one-hot manner due to a large number of possibilities. In such cases, an *n*-dimensional non-binary embedding is learned. However, with medical materials, we expect only a small number of categories for each application. Cross-entropy loss greatly simplifies the comparison problem for such cases, as no anchor input is needed. We believe this is viable because the problem space has been simplified—sample labels can only take two values (0 or 1). If one desires to learn a distance metric between a large number of medical material categories, the D-CNN could be tweaked to use triplet loss by adding an anchor input and changing the minimization objective.

Specifically, we train the D-CNN as follows. For a predetermined number of epochs, we train the network on a training set of patch comparison samples. At the end of each epoch, we then evaluate the network on a separate validation set of patch comparison samples. The loss on the validation set is tracked for each epoch, and if the current epoch’s validation set loss is the lowest of all epochs so far, the D-CNN model’s weights are saved. Ideally, the training regimen would converge to the lowest validation set loss on the final epoch, but this is not always the case.

Saving the lowest-loss D-CNN model rather than the final epoch D-CNN model mitigates risks of overfitting the model. Overfitting occurs when, in later epochs of the training process, the validation set loss increases due to a model losing its ability to generalize features learned from the training set. Our procedure avoids this by ignoring any D-CNN model iterations that yield a larger validation set loss than earlier epochs.

After training the network, the network is evaluated with a testing set of patch comparison samples it has not seen before. Like in training, the D-CNN makes binary similarity decisions between a reference patch and *n* comparison image patches. These similarity decisions are encoded in a *K*-dimensional vector p using Eq. 2.

$$p_{k}=\frac{1}{N_{k}}\underset{n|c_{n}=k}{\sum }s_{n}$$ (2)

The distance matrix D is built from the L2-norm between pairs of entries in p. Each entry in D, $d_{kk\prime }$, represents the perceptual distance the D-CNN has established between material categories *k* and k′. The value of each entry of D is presented in Eq. 3.

$$D_{kk\prime }={\left\|p_{k}-p_{k\prime }\right\|}_{2}$$ (3)

While training the D-CNN, we define the “optimal” D matrix as the one that is generated when the D-CNN has the lowest loss on the validation set. This optimal matrix is saved in addition to the model’s weights and is used as the basis for generating the material attributes in later steps.

### 2.3 Generating Material Attributes

The distance matrix D introduced in Section 2.2 maps distances from material categories to other material categories. However, we are also interested in discovering a set of *M* novel material attributes that provide new, useful information that can improve the categorization and separation of image patches.

We reintroduce the method in Schwartz and Nishino (2020) for mapping material categories to material attributes. This procedure preserves the distances discovered in D while introducing values for the mapping that reflect how humans generally perceive materials. This mapping is encoded in the *material category-attribute matrix* A.

A is a K×M matrix, where *K* is the number of material categories encoded by D and *M* is a freely selected value that defines the number of material attributes that are generated. The entries of A are bound to the range [0,1] so that each entry represents a conditional probability. The minimization objective for A is presented in Eq. 4.^2^

$$A^{\text{*}}=\underset{A}{\text{argmin}}\underset{k,k\prime \in C}{\sum }{\left(||a_{k}-a_{k\prime }|{|}_{2}-D_{kk\prime }\right)}^{2}$$ (4)

$$+\gamma \underset{p\in P}{\sum }\beta \left(p;a,b\right)\text{ln}\left(\frac{\beta \left(p;a,b\right)}{q\left(p;A\right)}\right)$$

$$q\left(p;A\right)=\frac{1}{KM}\underset{k,m}{\sum }{\left(2\pi h^{2}\right)}^{-\frac{1}{2}}\text{exp}\left(-\frac{{\left(a_{km}-p\right)}^{2}}{2h^{2}}\right)$$ (5)

The first term of the objective captures the distances between material categories in D and material attributes in A with a distance measure that iterates over the L2-distance of columns $a_{k}$ in A and compares them against individual entries in D.

The second term of the objective captures an important feature of the A matrix—that its entries should conform to a reasonable distribution that mirrors human perception. Like Schwartz and Nishino (2020), we use a beta distribution with parameters a,b=0.5. The beta distribution is ideal because, for human perception, material attributes usually either strongly exhibit a certain material category or not exhibit it at all. We assume that this observation, like with natural categories, holds with expert categories.

Since the Beta distribution is continuous, it still permits intermediate cases where materials may be similar (as is the case for “tumor” and “brain”). The γ-weighted term accomplishes this by embedding the A matrix in a Gaussian kernel density estimate q(p;A) and comparing it to the target beta distribution. This comparison is accomplished by evaluating the Kullback–Leibler (KL) divergence between those two terms. The Gaussian kernel density estimate of A at point *p* is presented in Eq. 5.

The optimized matrix $A^{*}$ from Eq. 4 is held constant and used as the A matrix in further portions of the system.

### 2.4 Material Attribute-Category Convolutional Neural Network Architecture

**FIGURE 3:**
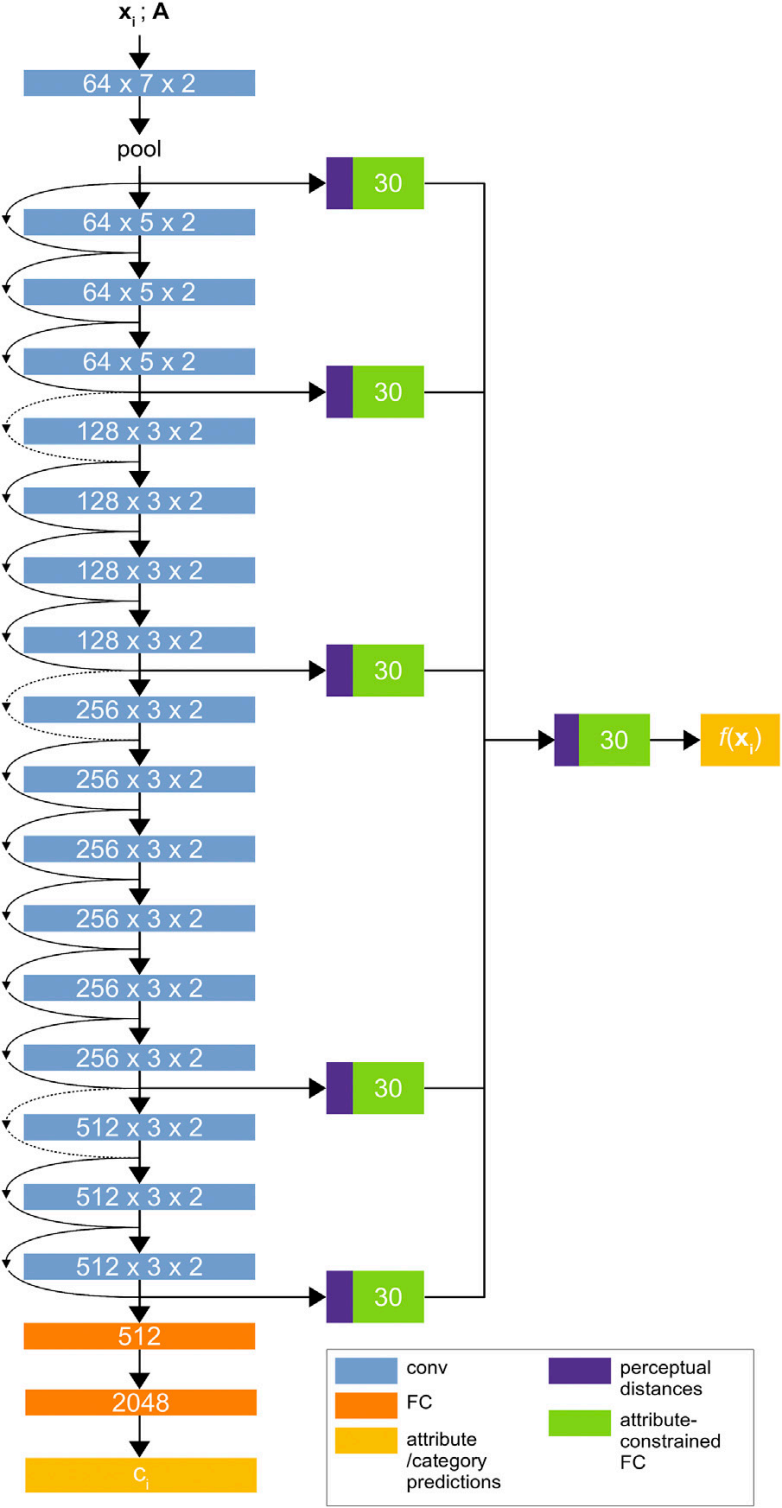
The Material Attribute Classifier CNN (MAC-CNN) architecture. The network uses convolutional layers from ResNet34 (He et al., 2015) followed by sequential 512-node and 2048-node fully connected layers to predict the material category $c_{i}\in {\left[0,1\right]}^{K}$. An auxiliary network of fully connected layers also predicts the material attribute probabilities $f\left(x_{i}\right)$.

The *material attribute-category convolutional neural network* (MAC-CNN) is an end-to-end convolutional neural network that seeks to directly learn the *K* material categories while also simultaneously learning the *M* material attributes embedded by A. We improve on the MAC-CNN design in Schwartz and Nishino (2020) by updating the architecture to classify medical materials more robustly. Figure 3 demonstrates the architecture of our MAC-CNN.

The MAC-CNN in Schwartz and Nishino (2020) used VGG-16 (Simonyan and Zisserman, 2014) as its backbone architecture. However, to maintain consistency with the D-CNN and use a more powerful architecture, we introduce an updated version of the MAC-CNN that is built on ResNet34 (He et al., 2015). ResNet is more reliable with deeper layers since its architecture reduces the vanishing gradient problem. This means that, when compared to a deeper version of the VGG network, a deeper version of ResNet could give the MAC-CNN greater predictive power, which could be useful for complex medical material problems. Like all models with more parameters, this comes at the expense of training time.

The fully-connected layers in the ResNet network are replaced by two fully-connected layers to be trained from random initialization. These layers determine the *K* material category predictions as shown in Figure 3, and output a one-hot vector with the material category classification. If the D-CNN is effective at discerning expert categories and the A matrix encodes these categories well, then the MAC-CNN should be able to categorize expert, naïve and null categories effectively.

To predict the *M* material attributes, the backbone network is augmented with multiple auxiliary classifier networks. The responses from each block of the ResNet backbone, along with the initial pooling layer, are used as inputs to individual auxiliary classifier networks. An additional auxiliary classifier is used to combine each module’s prediction into a single *M*-dimensional prediction vector. The auxiliary network learns to give conditional probabilities that the patch fits each material attribute, allowing the MAC-CNN to retain features that are informative for predicting material attributes.

The goal of the MAC-CNN is realized through training the network on image patches, like the D-CNN. However, the patches’ material categories are learned directly instead of through similarity decisions. The MAC-CNN also learns material attributes. Therefore, the weights from the D-CNN cannot be directly transferred to the MAC-CNN.

To predict the *M* discovered material attributes, the MAC-CNN uses a learned auxiliary classifier *f* with parameters Θ that maps an image patch with *d* raw features to the *M* attribute probabilities. The model *f*’s mapping is given by $f\left(x_{i};\Theta \right):{\mathbb{R}}^{d}\to {\left[0,1\right]}^{M}$. Each term in the output is a conditional probability that the patch exhibits that particular attribute.

Given a *D*-dimensional feature vector output from a hidden layer of the MAC-CNN, the *M* dimensional material attribute prediction is computed by Eq 6. The network’s weights and biases $\Theta =\left\{W_{1},W_{2},b_{1},b_{2}\right\}$ have dimensionality $W_{1}\in {\mathbb{R}}^{H\times D}$, $W_{2}\in {\mathbb{R}}^{M\times H}$, $b_{1}\in {\mathbb{R}}^{H}$, and $b_{2}\in {\mathbb{R}}^{M}$, where *H* is the dimensionality of the hidden layer.

$$f\left(x_{i};\Theta \right)=h\left(W_{2}h\left(W_{1}x_{i}+b_{1}\right)+b_{2}\right)$$

$$h\left(x\right)=\{\begin{array}{cc} 0 & x\leq 0\\ x & 0<x<1\\ 1 & x\geq 1 \end{array}$$ (6)

### 2.5 Material Attribute-Category Convolutional Neural Network Training

The convolutional layers in the backbone network are pretrained on ImageNet (Deng et al., 2009) for robust feature extraction, while the fully connected layers and auxiliary network are initialized with random weights. The training process optimizes these weights with respect to the target function and allows for a faster training process than starting with random weights for the entire network. A fast training process is important if the MAC-CNN is to be used in many different expert domains with little correlation to each other.

Like the D-CNN, we reduce overfitting by saving the MAC-CNN model from the training epoch with the lowest validation-set loss, which is not necessarily the model from the final epoch. This allows for the model to be trained for more epochs while mitigating potential overfitting later in the training process. To improve the MAC-CNN’s training convergence, we also use a learning rate scheduler that reduces the learning rate by a factor of 10 following epochs where validation set loss increases.

We train the network parameters Θ, dependent on the material attribute-category matrix A, to classify patches into *K* material categories and *M* material attributes simultaneously. The training set X is a set of *N* pairs of raw feature vectors and material category labels of the form $T=\left\{\left(x_{i},y_{i}\right)\right\}$, where $x_{i}$ is the raw feature vectors of image patch *i* and $y_{i}$ is a one-hot encoded label vector for its *K* material categories. Equation 7 formalizes the definition of these training pairs.

$$T=\left\{\left(x_{i},y_{i}\right):\;\;1\leq i\leq N,x_{i}\in {\mathbb{R}}^{d},\;y_{i}\in {\left\{0,1\right\}}^{K}\right\}.$$ (7)

The loss function and minimization objective for the MAC-CNN is given in Eq. 8, which follows from the loss function used in Schwartz and Nishino (2020).^3^ The loss function combines the negative log-likelihood of the *K* material category predictions for each image patch $x_{i}\in T$.

$$\Theta^{\text{*}}=\underset{\text{Θ}}{\text{argmin}}\underset{\left(x_{i},y_{i}\right)\in T}{\sum }\underset{\left(y_{j}\in y_{i}\right)}{\sum }-y_{j}\text{ln}\left(f{\left(x_{i};\Theta \right)}_{j}\right)$$

$$+\gamma_{1}\underset{p\in P}{\sum }\beta \left(p;a,b\right)\text{ln}\frac{\beta \left(p;a,b\right)}{q\left(p;f\left(T;\Theta \right)\right)}$$

$$+\gamma_{2}\overset{K}{\underset{k=1}{\sum }}||a_{k}-\frac{1}{\left|T_{k}\right|}\underset{\left(x_{i},y_{i}\right)\in T_{k}}{\sum }f\left(x_{i};\Theta \right)|{|}_{2}^{2}$$ (8)

The $\gamma_{1}$-weighted term represents the KL-divergence between the *M* material attribute predictions for $x_{i}$ and a Beta distribution with a,b=0.5. The Beta distribution is again chosen as a comparison distribution for reasons like those discussed in Section 2.2.

The $\gamma_{2}$-weighted term constrains the loss to the material attributes encoded in the A matrix. The term represents the mean squared error between rows of A, where each row represents one category’s probability distribution of attributes, and the material attribute predictions on the samples $T_{k}$ for each category.

The hyperparameters $\gamma_{1}$, $\gamma_{2}$ assign weights to their respective loss terms and are chosen at training time.

## 3 Results

The patch generation procedure, D-CNN, and MAC-CNN were implemented using the PyTorch neural network library (Paszke et al., 2019) and the Python programming language. The implementation was run on a system with an Intel Core i9 processor and two Nvidia Quadro RTX 8000 graphics cards. Our implementation is available on GitHub at https://github.com/cmolder/medical-materials.

To evaluate our methods on an expert domain, we compiled a dataset of local image patches of four categories—background, tumor, bone, and brain—using the procedure described in Section 2.1. These patches were generated from a combination of medical image datasets of knee X-rays and brain MRIs with tumors. The dataset was divided into a 60-20-20 percent training, validation, and testing split to be evaluated using our system.

### 3.1 Dataset

For bone category material patches, a set of 300 knee X-rays were sampled from the Cohort Hip and Cohort Knee (CHECK) baseline dataset (Bijlsma and Wesseling, 2015). For healthy brain and brain tumor category material patches, two datasets were combined: 3804 MRI scans with brain tumors were sourced from Cheng (2017) and additional brain MRI scans were sourced from The Cancer Imaging Archive (Clark et al., 2013; Schmainda and Prah, 2018).

These medical radiography scans were used to generate image patches using the procedure discussed in Section 2.1. The raw feature vectors from these image patches were then used to train, validate and test the D-CNN, optimize the material attribute-category matrix, and train, validate and test the MAC-CNN. 50 brain MRIs from Cheng (2017) were removed from the dataset to test the MAC-CNN’s capabilities of evaluating images in a sliding-window manner in Section 3.5.

The patches were generated using the process described in Section 2.1 at a size of 32×32 pixels.

### 3.2 Training Distance Matrix Convolutional Neural Network and Material Attribute-Category Convolutional Neural Network

To demonstrate that the D-CNN and MAC-CNN classifiers are trained effectively and do not overfit the training data, we present results from training multiple initializations of the D-CNN and MAC-CNN models. For reference, Table 2 contains the list of parameters we selected to train the D-CNN and MAC-CNN.

**TABLE 2:** D-CNN and MAC-CNN training parameters.

Notation	Definition	Value
**D-CNN**		
* E*	Number of epochs	15
* B*	Batch size	50
* η*	Learning rate	10^-3^
**MAC-CNN**		
* E*	Number of epochs	15
* B*	Batch size	50
* * $\eta_{0}$	Initial learning rate	10^-4^
* * $\gamma_{1}$	KL-divergence weight	10^-2^
* * $\gamma_{2}$	Perceptual difference weight	1

**FIGURE 4:**
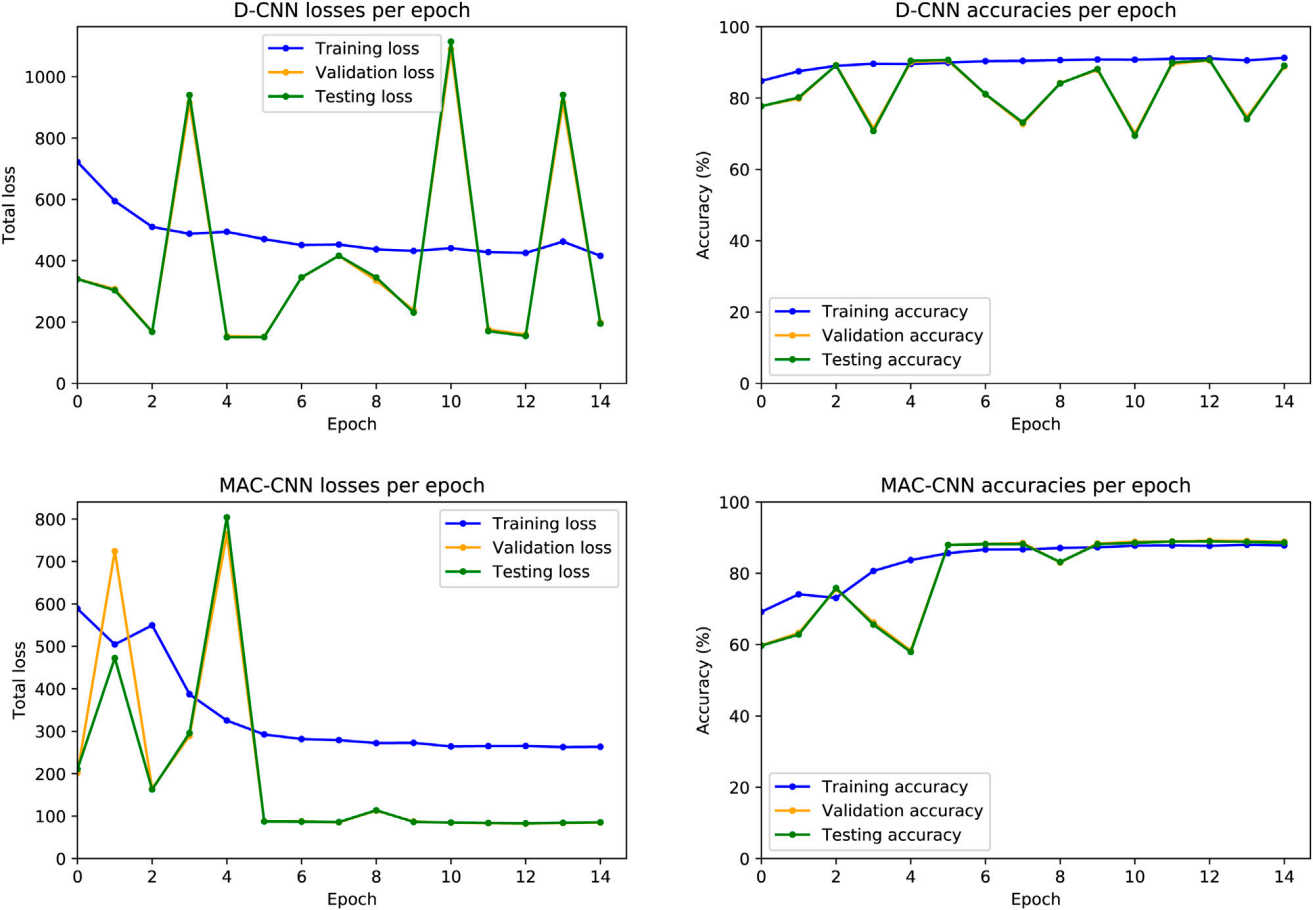
D-CNN and MAC-CNN loss and accuracies per epoch, for one randomly initialized pair of models. The training procedure always saves the model with the lowest validation set loss, regardless of whether it is from the last epoch. For the D-CNN in this example, the model from epoch 5 is saved, as well as the D matrix it generates on the validation set. For the MAC-CNN in this example, the model from the final epoch (epoch 14) is saved. Saving the lowest-loss model instead of the final model greatly mitigates the potential effects of overfitting in later epochs.

To evaluate how the training process affects the D-CNN and MAC-CNN, we first evaluated the effects of training a single instance of each network. For each network, we plotted the resulting loss and accuracy from each training epoch on the training, testing, and validation datasets. Figure 4 presents these results.

The resulting losses and accuracies yield three main findings—first, our decision to save the lowest-loss model rather than the final model is justified, especially for the D-CNN. For the D-CNN, validation and testing loss can vary significantly between epochs, and later epochs may yield noticeably higher losses and lower accuracies on the validation and testing sets. Second, testing and validation losses and accuracies trend very closely, as both sets are large and similar in size. Third, the learning rate scheduler used to train the MAC-CNN appears to better regulate its loss and accuracy in later epochs.

**FIGURE 5:**
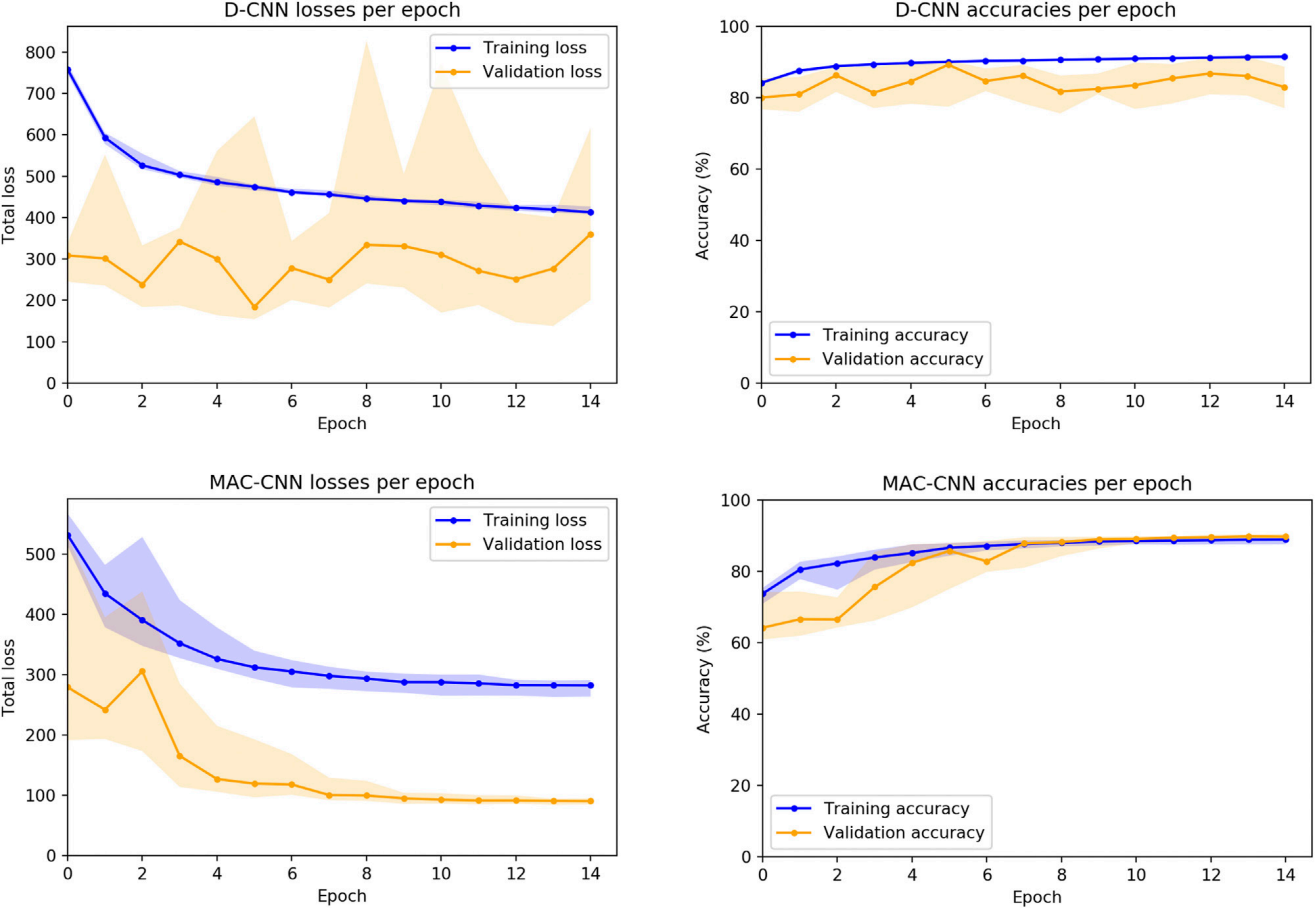
D-CNN and MAC-CNN loss and accuracies per epoch, sampled over 30 pretrained networks with random initializations of the fully connected layers. The lines represent the median loss/accuracy, and the shaded regions represent losses/accuracies between the 25th and 75th percentile for each epoch. As results from the validation and testing sets trend very closely, only results from the training and validation sets are presented.

We also considered how the random initializations of the non-ResNet34 layers affect the training of the networks. While the convolutional layers for both the D-CNN and MAC-CNN are initialized with weights pretrained on ImageNet (Deng et al., 2009), the fully connected and auxiliary layers are trained from scratch. Therefore, we trained 30 instances of both the D-CNN and MAC-CNN to see the loss and accuracy distributions on the training and validation sets.^4^ Figure 5 presents these distributions over the 15-epoch training process. The center lines depict the median loss and accuracy, while the shaded regions depict the region between the 25th and 75th percentiles of loss and accuracy.

The distribution plots demonstrate that the results in Figure 4 are typical of training a D-CNN and MAC-CNN. That is, the D-CNN trains more sporadically, but still achieves a lower validation loss during training, while the MAC-CNN trains more regularly and achieves its lowest validation loss in later epochs. The MAC-CNN is unlikely to overfit, as its validation loss does not typically increase late in the training process. The D-CNN has a somewhat greater risk of overfitting, but the impact of any potential overfitting from the D-CNN is mitigated by saving the lowest-loss model. It may be possible to regularize the D-CNN training by using a learning rate scheduler like the one used for the MAC-CNN.

As mentioned in Section 2.5, we would also like our models to have a short training time so they can be quickly applied to new expert medical domains. Therefore, we timed the training process of 10 instances of the D-CNN and MAC-CNN over 15 epochs. We found that the time required to train both the D-CNN and MAC-CNN, starting with pretrained convolutional layers, is relatively short.

In a separate experiment with a single, consumer-grade Nvidia RTX 2080 Ti graphics card, we evaluated the training times for 10 instances of the D-CNN and MAC-CNN using our implementation. Training 10 D-CNN instances for 15 epochs required an average of 23.7 min per instance (standard deviation 6.1 s), while training 10 ResNet34-based MAC-CNN instances for 15 epochs required an average of 14.3 min per instance (standard deviation 8.6 s).

### 3.3 Evaluating Distance Matrix Convolutional Neural Network Performance

On a testing set of 42,768 patches with evenly split categories, the D-CNN achieved an accuracy of 90.79%, which is the percentage of times that it correctly determined whether a reference and comparison patch were from the same material category or different material categories.

**FIGURE 6:**
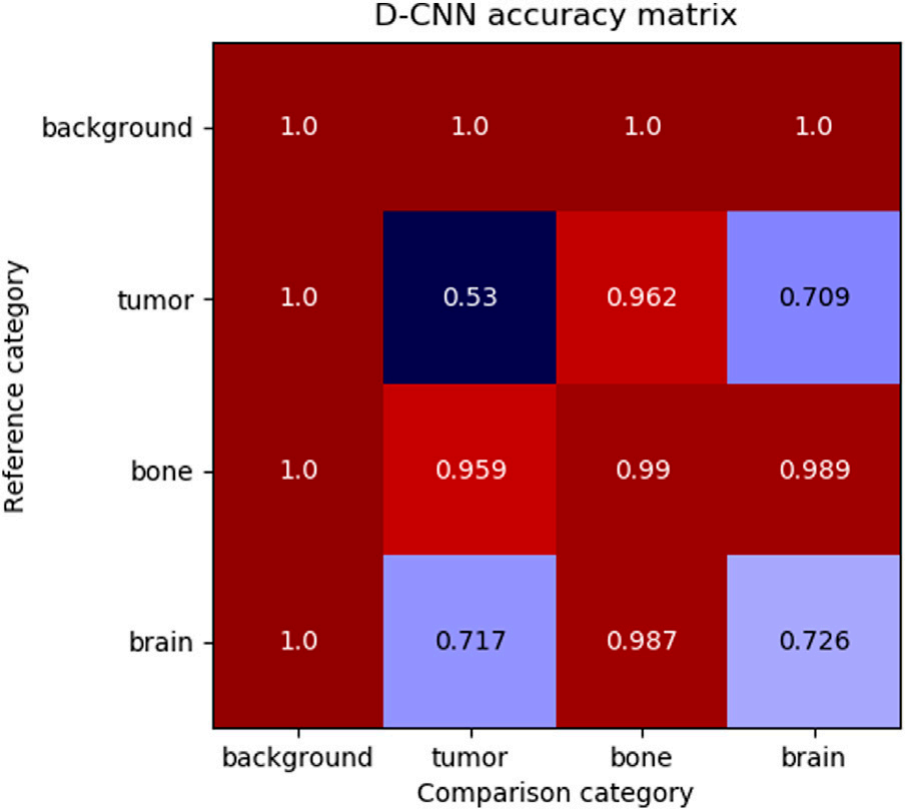
The accuracy of the D-CNN making correct similarity decisions between reference and comparison patches of every pair of categories. The null category, background, was easily determined to be similar or dissimilar to other patches due to its homogeneity and difference from other classes of patches. Meanwhile, the D-CNN was less accurate at classifying more similar pairs of categories, such as brain and tumor. The less accurate comparisons result in a smaller perceptual distance in the D matrix.

Although the D-CNN is accurate at making similarity decisions in general, the most informative accuracy values are those for each pair of material categories, as these accuracy values are reflective of the similarity between categories. Figure 6 demonstrates the accuracy of the D-CNN on each pair of category groupings.

These accuracies follow human intuition on how perceptually different these materials are expected to be. For example, brain and tumor patches generally appear similar, and therefore the D-CNN is less likely to correctly determine if patches of the two categories are the same or different. Meanwhile, the network is far more accurate at evaluating material patches that appear highly different, such as brain and bone.

### 3.4 Evaluating Material Attribute-Category Convolutional Neural Network Performance

The distance matrix generated during the training epoch where the D-CNN achieved the greatest validation accuracy was used as the basis for the material attribute-category matrix A. The L-BFGS-B algorithm optimized an A matrix with a minimal distance d(D,A) = 1.18.

Using this matrix, the MAC-CNN reached 92.82% accuracy at determining the material category of each image patch from a testing set. For reference, Schwartz and Nishino (2020) attained 84% accuracy at best for a given category. However, the fewer number of categories that our MAC-CNN is evaluating may make the classification problem easier, yielding a higher accuracy. Our network evaluates four categories while Schwartz and Nishino (2020) evaluated 13.

When withholding the material attributes and calculating loss as a mean squared error between the predicted and actual image patch material category, the accuracy of the MAC-CNN for determining material categories on the testing set was 91.74%. This shows that including the A matrix’s material attributes does not significantly alter the network’s ability to predict material categories.

Additionally, we compared the performance of our ResNet34-based MAC-CNN to a variant based on VGG-16 (Simonyan and Zisserman, 2014). The VGG-16 variant reflects the MAC-CNN architecture proposed by Schwartz and Nishino (2020), with convolutional sequential layers and an identical auxiliary network design. After training the VGG-16 model on the material patch dataset with the same learning parameters, the VGG-16 model had an accuracy of 93.39% for determining material categories on the testing set. This shows that the ResNet34 and VGG-16 models have comparable accuracy (within 0.6%). While these two smaller models perform similarly, ResNet’s better scalability to more layers (He et al., 2015) makes it advantageous for larger medical material datasets.

**FIGURE 7:**
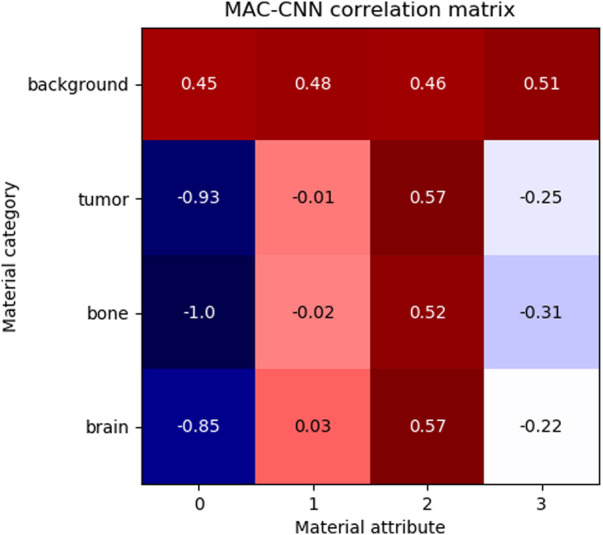
The correlation of MAC-CNN categorizations between material categories and material attributes. The most strongly exhibited association is with attribute 0 and the background category, which may be attributable to its homogeneity as the null category. Attributes 1 and 2 do not greatly separate the brain and tumor material categories, likely due to their small perceptual distance.

To evaluate the relationship between the material attributes learned by the MAC-CNN from A and the material categories of the image patches, a correlation matrix was generated to show how positively or negatively each learned material attribute related to the occurrence of the true label of a given material category. Figure 7 presents this matrix.

The matrix shows that attributes 1 and 3 are relatively uncorrelated to brain, bone, and tumor, and attribute 0 is negatively correlated to brain, bone, and tumor. Attribute 2 is moderately positively correlated with tumor and brain and slightly less positively correlated with bone. This matrix demonstrates that the attributes do not correspond one-to-one to given categories, meaning that the attributes encode different information than the categories.

An important factor in evaluating the MAC-CNN is determining if the material attributes encoded in A can accurately separate image patches by category. We used a method called *t*-SNE embedding (van der Maaten and Hinton, 2008), also used to evaluate the material attributes in Schwartz and Nishino (2020), to determine how well the MAC-CNN’s material attribute predictions separate material categories compared to the raw feature vectors of the patches. *t*-SNE embedding is a machine learning procedure that embeds the distributions of neighboring points in high-dimensional spaces to lower-dimension spaces, making the visualization of these high-dimensional spaces practical.

**FIGURE 8:**
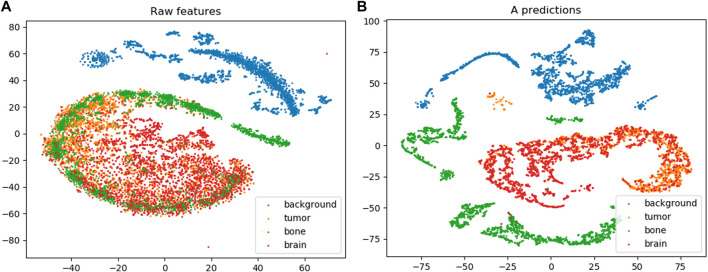
The results from t-SNE embedding (van der Maaten and Hinton, 2008) on the raw feature set **(A)** and the learned *M* attribute predictions encoded in A **(B)**. Although some separation is apparent on the raw feature embedding, especially between less-correlated categories like bone and brain, the separation is far stronger on the *M* attribute embedding, and even separates similar categories like brain and tumor while maintaining perceptual distances. This shows that the MAC-CNN has effectively learned to distinguished categories of image patches using the *M* attributes.

Figure 8 shows the *t*-SNE embedding on the raw feature vectors and the *M* attributes learned by the MAC-CNN from A on the test set. The graphs demonstrate a much clearer separation of categories for the material attributes compared to raw feature vectors, while also maintaining intuitive perceptual distances–for example, brain and tumor are more closely grouped than brain and bone. This indicates that the MAC-CNN’s learned attributes provide useful information that separates material patches by category compared to merely parsing the raw features.

### 3.5 Expanding Material Recognition to Full Images

As shown in Section 3.4, the MAC-CNN can accurately distinguish material categories from localized image patches. However, it is interesting and potentially useful to explore if this localized information can still yield useful results in the context of an entire image. If this were the case, then the MAC-CNN could be a promising component for future image analysis systems. However, it would not be reasonable to use the MAC-CNN alone since it is only able to extract *local* information, losing valuable information that comes from greater context.

To test the MAC-CNN to full medical scans, patches were sampled in a sliding-window fashion from full images. A 32×32 pixel window was used with a stride of 4 pixels.

**FIGURE 9:**
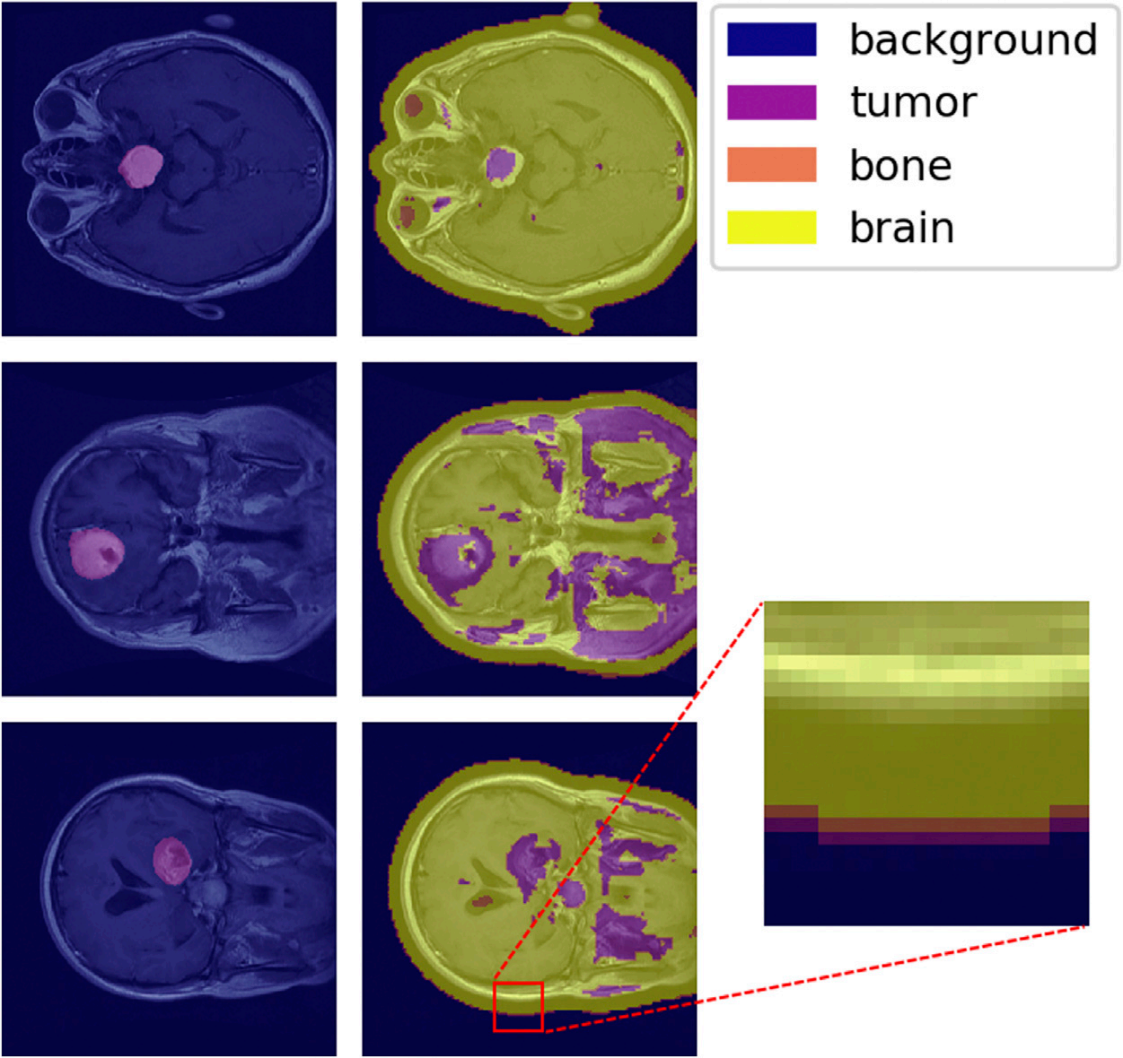
The MAC-CNN’s category decisions applied in a sliding-window manner to some full brain scans. The first column contains raw images with the expertly annotated mask (“tumor”) highlighted, while the second column contains raw images overlaid with the results from the MAC-CNN. The MAC-CNN is effective at detecting tumor regions, but often picks up extraneous noise. The network also appears to exhibit knowledge transfer from the knee X-rays, as it recognized bone textures around the perimeter of the skull that it learned from the knee X-rays.

The one-hot classification of material categories from performing a sliding-window analysis on the MAC-CNN was mapped to a matrix that contains the label of each patch sampled from the image. Figure 9 shows the MAC-CNN’s output on four brain MRI images using this convolutional system.

The MAC-CNN is effective in most cases at isolating the expertly annotated mask, which for the case of a brain scan is of the “tumor” category. However, the network is often too sensitive and miscategorizes some portions of the brain MRI as tumor despite it being outside the expertly annotated mask. The miscategorizations are likely because the network is only viewing small image patches of the MRI, meaning the network has no greater context when making its categorizations.

With that in consideration, the network still generally identified tumors when they were present. This shows that the network successfully learned a variety of textures that indicate the presence of a brain tumor. Interestingly, some transfer learning also occurred from learning on knee X-ray image patches, as the sliding-window analysis sometimes picked up the perimeter of the skull as having a bone texture. This shows that the MAC-CNN’s predictive power is robust enough to apply its categorizations from a variety of image types to other image types with similar textural appearances.

**FIGURE 10:**
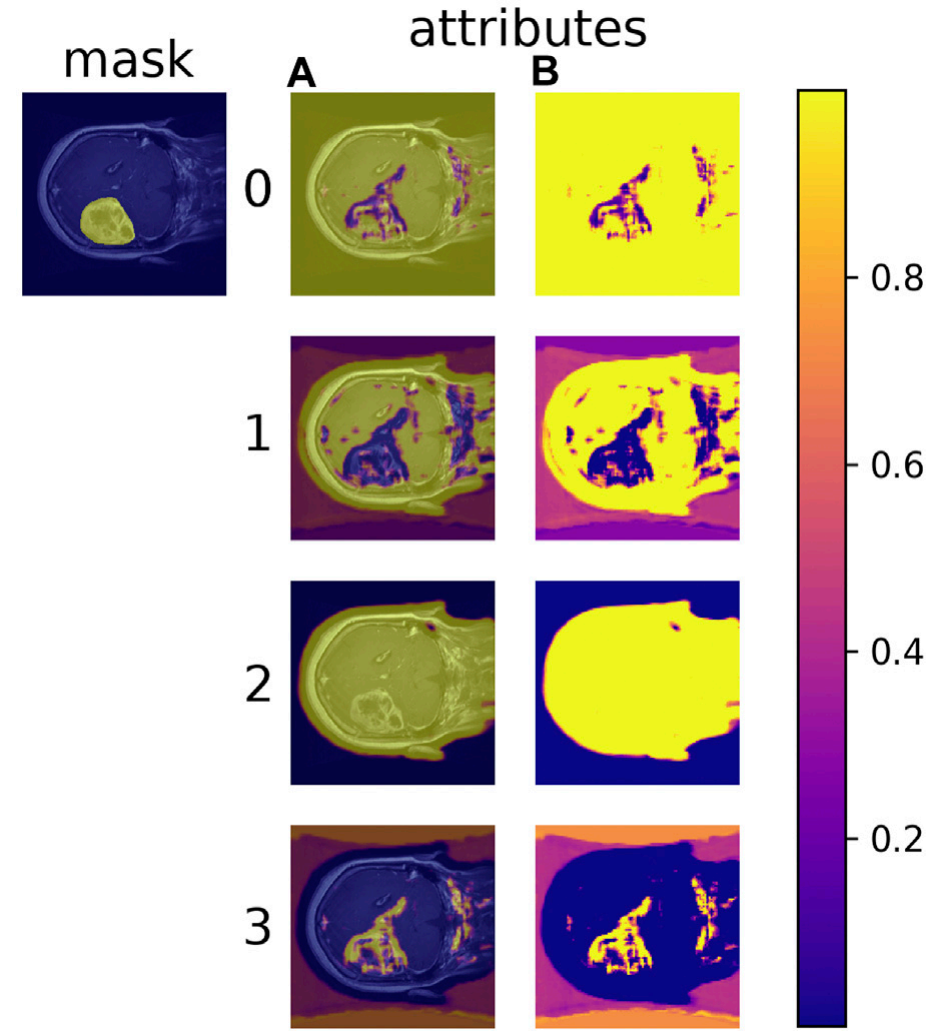
The MAC-CNN’s M=4 attributes applied in a sliding-window manner across a single brain scan. Each row represents a different attribute being evaluated, with the raw output **(B)** and the output overlaid on the image **(A)**. A higher value means the given attribute is expressed more strongly at that location in the image. Each attribute picks up different aspects of the scan, and different attributes can either positively or negatively exhibit aspects of different material categories. For example, attributes 0 and 1 negatively correlate to the expertly annotated mask (tumor) while attribute 3 correlates highly to it.

Learned material attributes may also provide insight into full image analysis. Figure 10 shows the MAC-CNN’s sliding-window evaluation of a single brain MRI on each of the *m* material attributes. The attributes appear to pick up different but useful information from the material categories. Attributes 0 and 1, for example, tend to identify regions of the scan that are *not* tumor, while attribute 3 tends to pick up on likely tumor regions. Meanwhile, attribute 2 tends to pick up regions that are non-null. This behavior tends to correspond to the correlation values presented in Figure 7.

## 4 Discussion

### 4.1 Related Work

Our methodology draws from many recent, relevant works about material analysis, computer vision, neural networks, and machine learning applications in medicine.

A few recent works in the medical field include applying machine learning to classify necrotic sections of pressure wounds (Zahia et al., 2018), segment brain scans (Lai et al., 2019), and segment chest X-rays (Wang et al., 2019).

In material analysis, there has been significant research into leveraging fully and weakly-supervised learning systems. Bell et al. (2015) introduced and evaluated the Materials in Context database, a large set of image patches with natural material category labels, in a fully supervised manner. Berg et al. (2010) proposed a weakly supervised attribute discovery model for data mining images and text on the Internet, which did include some local attribute classification. However, their network’s text annotations were associated with an entire image, and the images were not specific to an expert domain.

Material analysis has been performed in multiple expert domains with reduced data availability, including medicine. Gibert et al. (2015) performed material analysis on photographs of railroad tracks using several different domain-specific categories to detect decaying infrastructure. Their annotation uses a system of bounding-boxes on photographs of railroad ties to determine regions of given categories. Material analysis has also been studied in medicine. Marvasti et al. (2018) performed texture analysis on CT scans of liver lesions using a Bayesian network, evaluating features such as location, shape, proximity, and texture.

Specifically, our method is based on the material analysis method introduced by Schwartz and Nishino (2020). The work proposed a dataset of natural material categories and used a weakly supervised learning method to generate material attributes. The proposed method differs from Schwartz and Nishino (2020) in several ways. First, we specialize our method to medical radiography images, while Schwartz and Nishino (2020) focused exclusively on natural materials found in common photographs. Second, our method automatically generates a material distance metric from material patches using the D-CNN, while Schwartz and Nishino (2020) used human annotators to manually make binary similarity decisions among pairs of material patches. We decided this was necessary because the evaluation medical material similarity needs experts to properly evaluate by hand, and doctors and similar experts are scarce and expensive to retain in most situations. Third, our method upgrades the MAC-CNN proposed by Schwartz and Nishino (2020) by using the more scalable ResNet (He et al., 2015) architecture instead of VGG (Simonyan and Zisserman, 2014), letting larger, more augmented medical material datasets benefit from easier training on larger variants of the MAC-CNN.

We based the D-CNN on the Siamese neural network architecture as it has shown to be useful in a variety of similarity-evaluation problems. The Siamese neural network was first introduced by Bromley et al. (1994) to detect forgeries in digital signatures. Since then, Siamese networks have been used for human re-identification (Varior et al., 2016; Chung et al., 2017), one-shot image classification (Koch et al., 2015), object tracking (Bertinetto et al., 2016; Guo et al., 2017), and sentence similarity (Mueller and Thyagarajan, 2016). In medicine, Siamese networks have been used in similarity-evaluation tasks like gait recognition (Zhang et al., 2016), spinal metastasis detection (Wang et al., 2017), and to segment brain cytoarchitectonics (Spitzer et al., 2018).

Many novel neural network architectures have been proposed for computer vision tasks, including ResNeXt (Xie et al., 2017), DenseNet (Huang et al., 2017), PNASNet (Liu et al., 2018), and the Vision Transformer (ViT) (Dosovitskiy et al., 2020). For both the D-CNN and MAC-CNN, the ResNet (He et al., 2015) architecture is used. ResNet was selected over these other architectures for a few reasons.

For ViT, we do not believe the model is suitable for small texture patches. ViT divides its input into patches as tokens, and embeddings of these tokens are used as inputs into the model. While ViT achieves excellent performance on small-sized image datasets like CIFAR-10/100 (Krizhevsky, 2009), where each image is 32×32 pixels, such images contain more information than our texture patches. Each sub-patch of a CIFAR image sample may contain distinct information, but the sub-patches of a texture patch are not expected to do so because material patches only contain local context.

For PNASNet and other neural architecture search models, interpretability is sacrificed for accuracy. These discovered architectures are less interpretable than handcrafted architectures like ResNet. Interpretability is important in domains like medicine. For example, identifying causal relationships is key for doctors to diagnose conditions, and these causalities are easier to identify from interpretable models.

ResNet specifically has the following benefits. First, its structure, like VGG (Simonyan and Zisserman, 2014) and earlier convolutional architectures, allows for greater interpretability. The convolutional layers are stacked sequentially, and the feature maps of the hidden states can be visualized to determine what each convolutional filter detects. Second, unlike VGG, ResNet’s skip connections allow for the training of a much deeper network, which could be useful for complex, large medical material datasets with dozens of categories. Third, unlike some recent architectures, the purely sequential layers of ResNet’s design allow for an intuitive auxiliary network design. The sequential design allows for the auxiliary classifiers of the MAC-CNN to be placed so that each classifier processes a hidden state from a different stage of the network. With non-sequential models like ViT and PNASNet, finding an efficient placement of these auxiliary classifiers may be challenging. Fourth, ResNet models have a relatively small number of parameters compared to larger, more recent models, allowing for quicker training. This could be useful for specialized medical material problems, where a small group of researchers or doctors may not have many available computational resources.

U-Net (Ronneberger et al., 2015) uses a fully convolutional network to predict segmentation maps from input images. A fundamental difference between U-Net and the proposed method is that U-Net requires segmentation maps as ground truth label data. In the proposed method, we do not use segmentation maps as ground truth label data because often complete and complex segmentation maps are not available for training. For example, in Figure 9, to segment bone, brain tissue, brain tumor tissue, and the background, a 4-class segmentation map would be required by U-Net to be the label data for each training image. The dataset created in the proposed method instead uses a 2-class segmentation map: brain tumor tissue and everything else. In the proposed method, the dataset used to train the network uses class labels only or simple derived labels as explained in Section 2.1. The proposed method uses a patch generation process to create labeled material patches that can be used to train the network to pick up on local patterns relating to material type. This avoids the problem of expensive manual annotation.

### 4.2 Conclusion and Future Work

The D-CNN and MAC-CNN demonstrate that medical material categories can be successfully evaluated from radiography images using local information. They also demonstrate that naïve categories, such as healthy brain tissue in an MRI scan, are useful to augment expert categories, like brain tumors. We also demonstrated that such a system can be trained simultaneously on a range of expert, naïve, and null categories and can robustly pick up relevant categories without being conditioned on a subset of categories or attributes.

The knowledge transfer demonstrated on the brain MRIs and knee X-rays suggests that a larger version of the model would be able to analyze a more detailed or broader set of materials. For example, training this network on brain MRI data with more detailed labeling could yield greater accuracy and less noise than merely comparing healthy brain tissue and tumors. More granular data could also reduce the number of inaccurate predictions and noise when attempting sliding-window material categorization of whole images.

Rather than relying on more expensive segmentation maps to act as ground truth, instead, this model could be improved by modifying the patch generation procedure or sliding window approach. The patch generation procedure could be improved by iterating the process and using the model’s predictions to create a new set of patches, which can be used to train a new model. The sliding window approach uses a small, but fixed window size which makes it difficult to predict the labels of fine details in the image where multiple materials are present. The limitations of a fixed sliding window are avoided in U-Net (Ronneberger et al., 2015) at the cost of requiring complete segmentation maps for ground truth.

The D-CNN and MAC-CNN could also be extended to consider a larger context to further enhance material analysis. For example, a temporal dimension could be added to brain MRI data to model the progression of a brain tumor’s texture over time. Additionally, the networks could be extended to parse three-dimensional voxel data to extract more information from MRIs.

Overall, the D-CNN and MAC-CNN demonstrate the ability to perform expert material analysis from existing expertly annotated data without the need for experts to manually classify materials. The system also successfully demonstrates that intuitive observations about materials in nature can also hold in expert domains.

## Data Availability

The original contributions presented in the study are included in the article/Supplementary Material, further inquiries can be directed to the corresponding author. The datasets used to evaluate our models can be found in the following repositories: - Cohort Knee and Cohort Hip (CHECK): https://easy.dans.knaw.nl/ui/datasets/id/easy-dataset:62955 - brain tumor dataset: https://figshare.com/articles/brain_tumor_dataset/1512427 - Brain-Tumor-Progression: https://wiki.cancerimagingarchive.net/display/Public/Brain-Tumor-Progression The code used to implement our method and experiments can be found in the following GitHub repository: https://github.com/cmolder/medical-materials

## Footnotes

1 The D-CNN uses the Adam optimizer (Kingma and Ba, 2014) for gradient descent and weight updates.

2 The L-BFGS-B optimization algorithm is used to find a local minimum for the objective, starting from a randomized A with entries $a_{km}\in \left[0,1\right]$.

3 The MAC-CNN uses the Adam optimizer (Kingma and Ba, 2014) for gradient descent and weight updates.

4 The testing set loss and accuracy distributions are not included, as they have similar distributions to the validation set.
